# Karyotype asymmetry: again, how to measure and what to measure?

**DOI:** 10.3897/CompCytogen.v7i1.4431

**Published:** 2013-03-14

**Authors:** Lorenzo Peruzzi, Halil E. Eroğlu

**Affiliations:** 1 Dipartimento di Biologia, Università di Pisa, Via Luca Ghini 13, 56126 Pisa, Italy; 2 Bozok University, Faculty of Science and Art, Biology Department, 66200, Yozgat, Turkey

**Keywords:** Artificial chromosome datasets, chromosomal heterogeneity, karyotype asymmetry, asymmetry indices, interchromosomal asymmetry, intrachromosomal asymmetry, karyological parameters, Stebbins classification

## Abstract

One of the most popular, cheap and widely used approaches in comparative cytogenetics – especially by botanists – is that concerning intrachromosomal and interchromosomal karyotype asymmetry. Currently, there is no clear indication of which method, among the many different ones reported in literature, is the most adequate to infer karyotype asymmetry (especially intrachromosomal), above all in view of the criticisms recently moved to the most recent proposal published. This work addresses a critical review of the methods so far proposed for estimation of karyotype asymmetry, using both artificial and real chromosome datasets. It is shown once again how the concept karyotype of asymmetry is composed by two kinds of estimation: interchromosomal and intrachromosomal asymmetries. For the first one, the use of Coefficient of Variation of Chromosome Length, a powerful statistical parameter, is here confirmed. For the second one, the most appropriate parameter is the new Mean Centromeric Asymmetry, where Centromeric Asymmetry for each chromosome in a complement is easily obtained by calculating the difference of relative lengths of long arm and short arm. The Coefficient of Variation of Centromeric Index, strongly criticized in recent literature, is an additional karyological parameter, not properly connected with karyotype asymmetry. This shows definitively what and how to measure to correctly infer karyotype asymmetry, by proposing to couple two already known parameters in a new way. Hopefully, it will be the basic future reference for all those scientists dealing with cytotaxonomy.

## Introduction

Cytotaxonomy is a branch of cytogenetics, devoted to the comparative study of karyological features for systematic and evolutionary purposes (Siljak-Yakovlev and Peruzzi 2012). Today, a number of data can be obtained by chromosome studies: chromosome number, karyotype structure, karyotype asymmetry, chromosome banding, FISH, GISH and chromosome painting (Stace 2000, Levin 2002, Graphodatsky et al. 2011, Guerra 2012). Among them, one of the most popular, cheap and widely used approaches – especially by botanists – is that concerning karyotype asymmetry.

The concept of karyotype asymmetry, i.e. a karyotype marked by the predominance of chromosomes with terminal/subterminal centromeres (intrachromosomal asymmetry) and highly heterogeneous chromosome sizes (interchromosomal asymmetry), was developed for the first time by Levitsky (1931). Later, Stebbins (1971), in his masterpiece “*Chromosomal evolution in higher plants*”, proposed a quali-quantitative method for the estimation of karyotype asymmetry in twelve categories, by taking into account four classes (from 1 to 4), defined according to the increasing proportion of chromosomes with arm ratio <2:1, to be combined with three classes (from A to C) defined according to the increasing ratio between largest and smallest chromosome in a complement.

Concerning interchromosomal asymmetry, which is due to heterogeneity among chromosome sizes in a complement, other researchers proposed quantitative estimation methods in the following years. This is the case of the Rec index (Greilhuber and Speta 1976, Venora et al. 2002), the A_2_ index (Romero Zarco 1986), the R ratio (Siljak-Yakovlev 1996), the CV_CL_ (Lavania and Srivastava 1992, Watanabe et al. 1999, Paszko 2006). The latter, actually a Coefficient of Variation, is a statistically correct parameter and is able to capture even small variation among chromosome sizes in a complement. Hence, the estimation method for interchromosomal asymmetry does not need to be further discussed here.

More complex and debated is the quantitative estimation of the intrachromosomal asymmetry, which is due to centromere position. To address this issue, the first quantitative index proposed was the TF% of Huziwara (1962), soon followed by the AsK% of Arano (1963). Then, further proposals were AsI% of Arano and Saito (1980), Syi (Greilhuber and Speta 1976, Venora et al. 2002), A_1_ (Romero Zarco 1986), CG (Lavania and Srivastava 1992), A (Watanabe et al. 1999), CV_CI_ (Paszko 2006). The latter, a Coefficient of Variation of Centromeric Index, was claimed by Paszko (2006) to be the only parameter with statistical foundation. However, her proposal was recently strongly criticized by Zuo and Yuan (2011), who evidenced that CV_CI_ is not able to capture and quantitatively express the original meaning of karyotype asymmetry (i.e. the prevalence of telocentric-subtelocentric chromosomes), but only to quantify the relative variation (heterogeneity) among centromere positions in a karyotype. Hence, the problem of a correct intrachromosomal asymmetry estimation is still open.

Finally, a few authors tried to combine the two kinds of asymmetry in a single index, such as Lavania and Srivastava (1992) with DI, Paszko (2006) with AI. However, both these indices were strongly criticized, by Paszko (2006) and Peruzzi et al. (2009) respectively, and their use has to be definitely discouraged. The aim of this review is to critically analyze the proposed methods for estimating intrachromosomal asymmetry and to elaborate the proposal for a new suitable estimator which should be: 1) strictly quantitative, 2) statistically correct, 3) not a dispersion or variability index.

## Which kind of basic measures were used – and differently combined – for Intrachromosomal Asymmetry estimation?

Fundamentally, the basic measures, used in every method proposed so far, are those concerning the length of long (L) and short arm (S) of each chromosome in a complement. All the karyotypes where these measures are not applicable (for instance those with holocentric chromosomes or those with very small chromosomes, 1 µm or less), are not suitable for the estimation of intrachromosomal asymmetry at all. For all the others (the majority), typically L ≥ S ≥ 0 and L ≥ S. The variation extremes are S = L (i.e. chromosomes with centromere perfectly median) and S = 0 (i.e. chromosomes with centromere perfectly terminal). These two variables were combined by researchers in various ways:

**L/S** also called ***arm ratio* (r)**, it was used for instance in the widely known chromosome nomenclature proposed by Levan et al. (1964). Its values can range from **1** (if S = L) to **+∞** (the limit for S = 0).

**S/L** first proposed by Battaglia (1955), it is reciprocal to the arm ratio. Its values can range from **1** (if S = L) to **0** (if S = 0). It is fundamentally used also in **Syi** = (Mean S length / Mean L length) × 100 (Greilhuber and Speta 1976, Venora et al. 2002).

**S/(L+S)** also called ***centromeric index***, it is the proportion of short arm respect with the whole chromosome. Its values can range from **0.5** (if S = L) to **0** (if S = 0). It is fundamentally used in **TF%** = Total length of S in a chromosome set / Total length of a chromosome set × 100 (Huziwara 1962), **CG** = Median S length / Median (L+S) length × 100 (Lavania and Srivastava 1992), and **CV_CI_** (Paszko 2006).

**L/(L+S)** it is the proportion of long arm respect with the whole chromosome, being complementary to the centromeric index. Indeed, [L/(L+S)] + [S/(L+S)] = 1. Its values can range from **0.5** (if S = L) to **1** (if S = 0). It is fundamentally used in **AsK%** = Total length of L in a chromosome set / Total length of a chromosome set × 100 (Arano 1963) and the identical **AsI%** (Arano and Saito 1980).

**(L–S)/L** it was conceived in order to be complementary to S/L, indeed [(L–S)/L]+S/L = 1. Its values can range from **0** (if S = L) to **1** (if S = 0). It is used in **A_1_** = 1 – Mean S/L (Romero Zarco 1986).

**(L–S)/(L+S)** it is the difference between the two (complementary) proportions L/(L+S) and S/(L+S). Hence, its values can range from **0** (if S = L) to **1** (if S = 0). It is used in **A** = Mean (L–S)/(L+S) (Watanabe et al. 1999). *Please note that it can be expressed also as 2L/(L+S) – 1 or as 1 – 2S/(L+S)*.

Given that L/(L+S) and S/(L+S) are the only parameters which are formally correct on descriptive statistical grounds (they are both proportions, or relative lengths), and given their peculiar complementary relationships, the only parameter well suited to capture the mean intrachromosomal asymmetry in a karyotype is that proposed by Watanabe et al. (1999). It is noteworthy that these authors already stressed that their method is preferable respect with others “*because it usually follows a normal distribution*”. Indeed, given an artificial dataset of chromosomes with normal distribution (mean = median), only the estimators L/(L+S), S/(L+S) and their difference (L–S)/(L+S) are able to correctly describe these features (Table 1). However, it also must be noted that all the other karyotype intrachromosomal asymmetry estimators proposed in literature (Syi, TF%, CG, AsK%, A_1_), albeit not statistically correct, are highly correlated with A, with values typically above r = |0.9|, p < 0.01 (Paszko 2006, Peruzzi et al. 2009).

**Table 1.** Comparison of different estimators of intrachromosomal asymmetry on a set of 11 artificial chromosomes with gradually increasing asymmetry, from perfectly median (on the left) to perfectly terminal (on the right) centromeres. Also the mean values are reported in the last column on the right. L/S was excluded because no real value is obtained when S = 0.

	**chromosome**											
	**1**	**2**	**3**	**4**	**5**	**6**	**7**	**8**	**9**	**10**	**11**	**mean**
**S (µm)**	10	9	8	7	6	**5**	4	3	2	1	0	**5**
**L (µm)**	10	11	12	13	14	**15**	16	17	18	19	20	**15**
**S/L**	1.00	0.82	0.67	0.54	0.43	0.33	0.25	0.18	0.11	0.05	0.00	0.40
**S/(L+S)**	0.50	0.45	0.40	0.35	0.30	0.25	0.20	0.15	0.10	0.05	0.00	**0.25**
**L/(L+S)**	0.50	0.55	0.60	0.65	0.70	0.75	0.80	0.85	0.90	0.95	1.00	**0.75**
**(L-S)/L**	0.00	0.18	0.33	0.46	0.57	0.67	0.75	0.82	0.89	0.95	1.00	0.60
**(L-S)/<br/> (L+S)**	0.00	0.10	0.20	0.30	0.40	**0.50**	0.60	0.70	0.80	0.90	1.00	**0.50**

## How to compare Karyotype Asymmetry among individuals, populations, species etc.?

Let us return to karyotype asymmetry as a whole, with its two parts: interchromosomal and intrachromosomal. Concerning the measure of interchromosomal asymmetry, as explained above, the main point is to measure how much the chromosome lengths of a complement are different each other, and CV_CL_ (Paszko 2006) is perfectly suited for it. As all coefficients of variation, it is a ratio between standard deviation and mean of a sample (i.e. a dispersion index) × 100 (Sokal and Rohlf 1981). Typically, this parameter ranges from 0 (no variation) to 100 or more (in those cases of exceptionally heterogeneous samples, where standard deviation can be higher than the mean).

Concerning the measure of intrachromosomal asymmetry, CV_CI_ should not be used for the reasons explained above. Indeed, it is actually a measure of intrachromosomal heterogeneity, which does not necessarily means asymmetry in the original sense given by Levitsky (1931) and Stebbins (1971). Among others, as shown above, the statistically best suited parameter is A (Watanabe et al. 1999), ranging from 0 (perfectly symmetric) to 1 (perfectly asymmetric).

Since the two kinds of asymmetry express different concepts, it is not desirable to combine them in a single value. On the contrary, as argued for the first time by Romero Zarco (1986) and then by Peruzzi et al. (2009), the best way in representing karyotype asymmetry relationships among organisms is by means of bidimensional scatter plots, where the two asymmetry estimators are put in the x and y axes and points represent each sample. Up to the present day, this was done with the couples of parameters A_1_ and A_2_ (Romero Zarco 1986) or CV_CI_ and CV_CL_ (noteworthy, CV_CL_ = A_2_ × 100) (Paszko 2006; Peruzzi et al. 2009).

**Figure 1.**
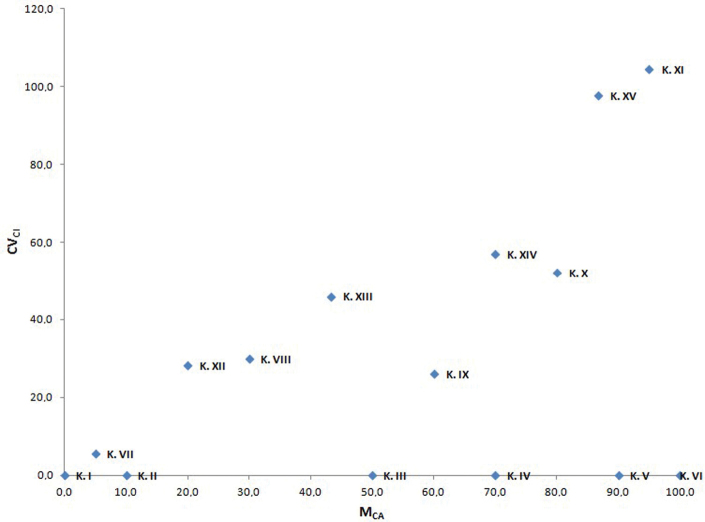
Scatter plot of the fifteen artificial karyotypes reported in Table 2 against M_CA_ (x axis) and CV_CI_ (y axis).

**Figure 2.**
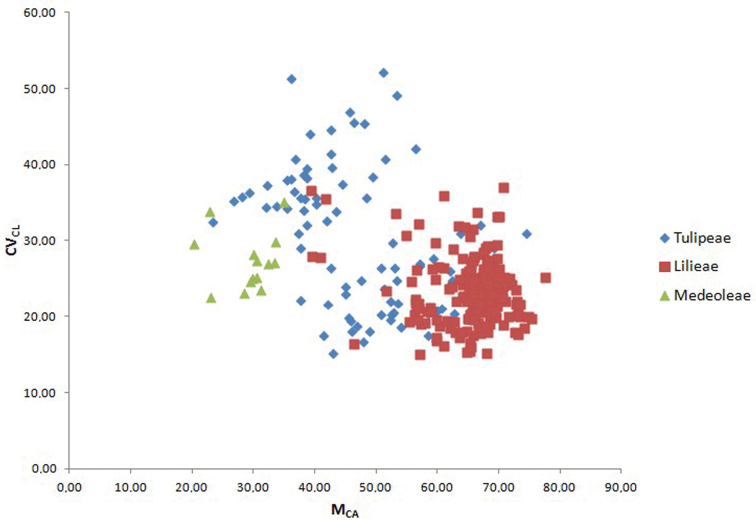
Scatter plot of samples from the three tribes Medeoleae, Tulipeae and Lilieae against M_CA_ (x axis) and CV_CL_ (y axis). Data derived from the dataset published by Peruzzi et al. (2009).

The present proposal is to couple CV_CL_ with a new parameter called **M_CA_ (Mean Centromeric Asymmetry)**, where Centromeric Asymmetry of a single chromosome is given by the formula (L-S)/(L+S). Accordingly, M_CA_ = A × 100. Generally, CV_CI_ is not correlated with M_CA_ (e.g. in small dataset of *Calamagrostis* Adanson, 1753 used by Paszko 2006), so that it could be used sometimes as an optional third parameter to reveal karyotype relationships among organisms, besides asymmetry *sensu stricto*. This could be useful especially when chromosome size variation is negligible. For instance, in an artificial karyotypes dataset with no chromosome size variation (Table 2), it is once again evident how samples, even with the most different intrachromosomal asymmetries (M_CA_), could not be discriminated by CV_CL_ if there is not variation in chromosome size. Conversely, samples with almost identical intrachromosomal asymmetry can reveal their different karyotype structure following use of the CV_CI_ (compare karyotypes III and XIII, or IV and XIV, in Figure 1). In some special case, if CV_CI_ results positively correlated with M_CA_, this former additional karyological parameter is not useful at all and may be omitted. This is the case of the large Liliaceae dataset used by Peruzzi et al. (2009), where the correlation among CV_CI_ and M_CA_ is r = 0.792 (p < 0.01). As can be seen in Figure 2, the three tribes of subfamily Lilioideae show a clear tendency to have karyotypes distinct on asymmetry grounds: tribe Medeoleae, with relatively low intrachromosomal (M_CA_) and interchromosomal asymmetry (CV_CL_), tribe Tulipeae, with higher interchromosomal asymmetry, and tribe Lilieae, with higher intrachromosomal asymmetry. Almost identical results were indeed obtained by Peruzzi et al. (2009) by using the, now “old-fashioned”, couple CV_CI_ and CV_CL_. Finally, it is also important to remember here, once again, that a symmetric karyotype does not necessarily implies “primitivity”, as assumed by earlier students (see, for instance, Siljak-Yakovlev 1996 for the concept of “secondary symmetry”). As for other cytotaxonomic features, once karyological relationships between taxa are demonstrated, it is also important to have some independent source of information in order to infer the direction of changes (Siljak-Yakovlev and Peruzzi 2012).

**Table 2.** Karyomorphometric features of a dataset with fifteen artificial karyotypes, all with the chromosomes of the same length (no chromosome size variation).

chromosome																								
	1		2		3		4		5		6		7		8		9		10		11		12	
karyotype	L	S	L	S	L	S	L	S	L	S	L	S	L	S	L	S	L	S	L	S	L	S	L	S
karyotype I	5.0	5.0	5.0	5.0	5.0	5.0	5.0	5.0	5.0	5.0	5.0	5.0	5.0	5.0	5.0	5.0	5.0	5.0	5.0	5.0	5.0	5.0	5.0	5.0
karyotype II	5.5	4.5	5.5	4.5	5.5	4.5	5.5	4.5	5.5	4.5	5.5	4.5	5.5	4.5	5.5	4.5	5.5	4.5	5.5	4.5	5.5	4.5	5.5	4.5
karyotype III	7.5	2.5	7.5	2.5	7.5	2.5	7.5	2.5	7.5	2.5	7.5	2.5	7.5	2.5	7.5	2.5	7.5	2.5	7.5	2.5	7.5	2.5	7.5	2.5
karyotype IV	8.5	1.5	8.5	1.5	8.5	1.5	8.5	1.5	8.5	1.5	8.5	1.5	8.5	1.5	8.5	1.5	8.5	1.5	8.5	1.5	8.5	1.5	8.5	1.5
karyotype V	9.5	0.5	9.5	0.5	9.5	0.5	9.5	0.5	9.5	0.5	9.5	0.5	9.5	0.5	9.5	0.5	9.5	0.5	9.5	0.5	9.5	0.5	9.5	0.5
karyotype VI	10	0	10	0	10	0	10	0	10	0	10	0	10	0	10	0	10	0	10	0	10	0	10	0
karyotype VII	5.0	5.0	5.0	5.0	5.0	5.0	5.0	5.0	5.0	5.0	5.0	5.0	5.5	4.5	5.5	4.5	5.5	4.5	5.5	4.5	5.5	4.5	5.5	4.5
karyotype VIII	5.5	4.5	5.5	4.5	5.5	4.5	5.5	4.5	5.5	4.5	5.5	4.5	7.5	2.5	7.5	2.5	7.5	2.5	7.5	2.5	7.5	2.5	7.5	2.5
karyotype IX	7.5	2.5	7.5	2.5	7.5	2.5	7.5	2.5	7.5	2.5	7.5	2.5	8.5	1.5	8.5	1.5	8.5	1.5	8.5	1.5	8.5	1.5	8.5	1.5
karyotype X	8.5	1.5	8.5	1.5	8.5	1.5	8.5	1.5	8.5	1.5	8.5	1.5	9.5	0.5	9.5	0.5	9.5	0.5	9.5	0.5	9.5	0.5	9.5	0.5
karyotype XI	9.5	0.5	9.5	0.5	9.5	0.5	9.5	0.5	9.5	0.5	9.5	0.5	10	0	10	0	10	0	10	0	10	0	10	0
karyotype XII	5.0	5.0	5.0	5.0	5.0	5.0	5.0	5.0	5.5	4.5	5.5	4.5	5.5	4.5	5.5	4.5	7.5	2.5	7.5	2.5	7.5	2.5	7.5	2.5
karyotype XIII	5.5	4.5	5.5	4.5	5.5	4.5	5.5	4.5	7.5	2.5	7.5	2.5	7.5	2.5	7.5	2.5	8.5	1.5	8.5	1.5	8.5	1.5	8.5	1.5
karyotype XIV	7.5	2.5	7.5	2.5	7.5	2.5	7.5	2.5	8.5	1.5	8.5	1.5	8.5	1.5	8.5	1.5	9.5	0.5	9.5	0.5	9.5	0.5	9.5	0.5
karyotype XV	8.5	1.5	8.5	1.5	8.5	1.5	8.5	1.5	9.5	0.5	9.5	0.5	9.5	0.5	9.5	0.5	10	0	10	0	10	0	10	0
